# Molecular genetics of pulmonary hypertension in children

**DOI:** 10.1016/j.gde.2022.101936

**Published:** 2022-08

**Authors:** Fatima Taha, Laura Southgate

**Affiliations:** Molecular and Clinical Sciences Research Institute, St George’s University of London, London, UK

## Abstract

Until recently, the molecular aetiology of paediatric pulmonary hypertension (PH) was relatively poorly understood. While the TGF-β/BMP pathway was recognised as central to disease progression, genetic analyses in children were largely confined to targeted screening of risk genes in small cohorts, with clinical management extrapolated from adult data. In recent years, next-generation sequencing has highlighted notable differences in the genetic architecture underlying childhood-onset cases, with a higher genetic burden in children partly explained by comorbidities such as congenital heart disease. Here, we review recent genetic advances in paediatric PH and highlight important risk factors such as dysregulation of the transcription factors SOX17 and TBX4. Given the poorer prognosis in paediatric cases, molecular diagnosis offers a vital tool to enhance clinical care of children with PH.

**Current Opinion in Genetics & Development** 2022, **75**:101936

This review comes from a themed issue on **Molecular and genetic basis of disease**

Edited by **Neil Hanchard** and **Heather Mefford**

For complete overview of the section, please refer to the article collection, “Molecular and Genetic Basis of Disease”

Available online 27th June 2022

https://doi.org/10.1016/j.gde.2022.101936

0959-437X/© 2022 The Author(s). Published by Elsevier Ltd. This is an open access article under the CC BY license (http://creativecommons.org/licenses/by/4.0/).

## Introduction

Pulmonary arterial hypertension (PAH) is a rare, progressive and usually fatal disorder with an estimated annual incidence of 4–10 cases/million and a prevalence of 20–40 cases/million in children [1]. It is defined as a mean pulmonary arterial pressure>20 mmHg and pulmonary vascular resistance ≥3 Wood Units (WU) [2]. Paediatric PAH is often associated with a more severe clinical course, particularly in the presence of other congenital defects. However, early treatment in children can significantly improve overall outcomes resulting in transplant-free 5-year survival rates over 80%, depending on PAH aetiology and WHO functional class at diagnosis 3, 4, 5.

**Figure 1:**
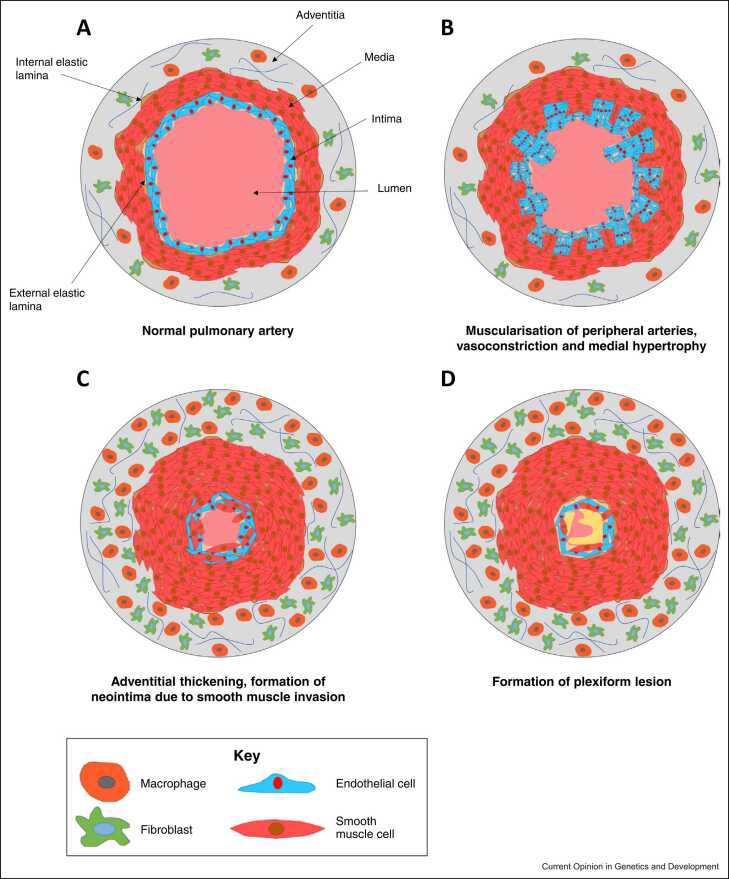
Pathobiology of PAH. **(a)** Cross-sectional representation of a healthy pulmonary artery. **(b)** Early stages of PAH include vasoconstriction due to pulmonary endothelial dysfunction and medial hypertrophy. **(c)** Progressive vascular remodelling of distal pulmonary arteries is characterised by proliferation and migration of smooth muscle cells, endothelial cells, and fibroblasts. **(d)** Plexiform lesions, due to endothelial dysfunction, proliferation and apoptotic resistance are a hallmark feature of late-stage PAH.

PAH has a complex aetiology with different factors precipitating disease presentation in adult- and childhood-onset disease, resulting in a varied pathobiology, disease severity and genetic background (Figure 1). The disorder is classified into several subgroups, which include idiopathic PAH (IPAH), heritable PAH (HPAH) and associated forms PAH (APAH) (Table 1). In childhood-onset disease, the vast majority of patients are diagnosed with IPAH, HPAH or PAH associated with congenital heart disease (APAH-CHD) [6].

**Table 1:** Clinical classification of PH.

Group 1	PAH
1.1	IPAH
1.2	HPAH
1.3	Drug- and toxin-induced PAH
1.4	APAH
* 1.4.1*	*PAH associated with connective tissue disease (APAH-CTD)*
* 1.4.2*	*PAH associated with human immunodeficiency virus infection*
* 1.4.3*	*PAH associated with portal hypertension*
* 1.4.4*	*APAH-CHD*
* 1.4.5*	*PAH associated with schistosomiasis*
1.5	PAH long-term responders to calcium channel blockers
1.6	PAH with overt features of venous/capillaries (PVOD/PCH) involvement
1.7	PPHN syndrome
Group 2	PH due to left heart disease
2.1	PH due to heart failure with preserved left ventricular ejection fraction
2.2	PH due to heart failure with reduced left ventricular ejection fraction
2.3	Valvular heart disease
2.4	Congenital/acquired cardiovascular conditions leading to post-capillary PH
Group 3	PH due to lung diseases and/or hypoxia
3.1	Obstructive lung disease
3.2	Restrictive lung disease
3.3	Other lung disease with mixed restrictive/obstructive pattern
3.4	Hypoxia without lung disease
3.5	Developmental lung disorders
Group 4	PH due to pulmonary artery obstructions
4.1	Chronic thromboembolic pulmonary hypertension
4.2	Other pulmonary artery obstructions
Group 5	PH with unclear and/or multifactorial mechanisms
5.1	Haematological disorders
5.2	Systemic and metabolic disorders
5.3	Others
5.4	Complex CHD

Updated clinical classification according to the sixth World Symposium on Pulmonary Hypertension [2]. Group 1 comprises subgroups of PAH, within which IPAH (1.1), HPAH (1.2) and APAH-CHD (1.4.4) are most common in children.

In this review, we summarise the key genes and pathways dysregulated in paediatric PAH with a focus on recent advances that highlight the applicability of molecular insights to clinical management.

## BMP/TGF-β signalling in paediatric pulmonary arterial hypertension

### Dysregulation of the canonical BMP/TGF-β pathway

The significance of canonical BMP/TGF-β signalling in the pathogenesis of both adult and paediatric PAH has been well documented 7, 8. Following the identification of *BMPR2* haploinsufficiency as a molecular mechanism of disease, additional causal variants were described in genes encoding the ALK1 (*ACVRL1*) and Endoglin (*ENG*) receptors, which have been observed in association with hereditary haemorrhagic telangiectasia (HHT). By contrast, initial reports of pathogenic variation in the type I receptor ALK6 (*BMPR1B*) have not been robustly validated, indicating a limited role for this gene in paediatric PAH 7, 8, 9• (Table 2).

**Table 2:** Summary of main clinical findings associated with genetic variants in paediatric PAH.

Gene (protein) symbol	OMIM reference	PAH group (%)	Inheritance pattern	Associated clinical features	Probands reported (*n*)	Age of diagnosis (y)	mPAP (mmHg)	PVR (WU) or PVRi (WU m^2^)	mPWP (mmHg)	CI (L/min/m^2^)	Drug response	Recent key references
*ABCC8* (SUR1)	600509	IPAH (62%); APAH-CHD (23%); HPAH (15%)	AD	>Gene also linked to familial hyperinsulinaemic hypoglycaemia, transient/permanent neonatal diabetes mellitus >A nonsense variant has been reported in a patient with PPHN and hypoglycaemia	13	8.0 ± 6.0 (*n* = *11*)	51.2 ± 8.0 (*n* = *5*)	20.1 ± 6.6 WU m^2^ (*n* = *5*)	8.0 ± 0 (*n* = *3*)	2.8 ± 0.61 (*n* = *4)*	>Activation of SUR1 may be a potential therapeutic target for PAH >Diazoxide may resolve symptoms in adults but can lead to PH in hypoglycaemic infants	29, 30, 63•
*ACVRL1* (ALK1)	601284	PAH-HHT (44%); IPAH (38%); HPAH (18%)	AD	HHT type 2 (epistaxis, mucocutaneous telangiectases, visceral AVMs)	40	8.2 ± 5.4 (*n* = *40*)	67.0 ± 17.8 (*n* = *30*)	22.4 ± 12.5 WU (*n* = *12*); 20.0 ± 12.2 WU m^2^ (*n* = *16*)	9.0 ± 3.6 (*n* = *23*)	3.2 ± 1.0 (*n* = *28*)	96% (23/24) have no response to acute vasodilator test	10, 64
*ATP13A3*	610232	HPAH (50%); IPAH (25%); APAH-CHD (25%)	AD; AR	>Biallelic variants are linked to a severe, early-onset form of PAH >A heterozygous variant has been reported in one child with PAH and secundum ASD	4	2.7 ± 3.0 (*n* = *6*)	46 ± 9.7 (*n* = *4*)	19.3 ± 10.6 WU (*n* = *3*)	nd	1.7 (*n* = *1*)	>Biallelic variants are associated with rapidly progressive refractory PAH >Pre-emptive Potts shunt has been used successfully in one case	21, 23, 65
*BMP10*	608748	IPAH (50%); APAH-CHD (50%)	AD	–	2	7.0 ± 4.0 (*n* = *2*)	35 (*n* = *1*)	nd	nd	3.5 (*n* = *1*)	–	17•, 18, 21
*BMPR1B*	603248	IPAH (75%); APAH-CHD (25%)	AD	>Gene also linked to brachydactyly >One paediatric case reported with PAH and ASD	4	10.3 ± 2.7 (*n* = *4*)	88.5 ± 22.5 (*n* = *2*)	nd	9 (*n = 1*)	2.8 ± 0.8 (*n* = *2*)	–	–
*BMPR2*	600799	IPAH (51%); HPAH (39%); APAH-CHD (10%)	AD	–	>130	10.2 ± 5.0 (*n* = *109*)	69.8 ± 15.0 (*n* = *31*)	19.0 ± 10.8 WU (*n* = *6*); 22.1 ± 11.9 WU m^2^ (*n* = *21*)	9.4 ± 2.6 (*n* = *20*)	3.2 ± 1.4 (*n* = *24*)	75% (12/16) have no response to acute vasodilator test	10, 16, 56
*CAV1*	601047	HPAH (60%); IPAH (20%); APAH (20%)	AD	>Gene also linked to congenital lipodystrophies >A *de novo* variant has been reported in one case of neonatal lipodystrophy, PAH and PAVM	5	4.2 ± 3.0 (*n* = *5*)	50.5 ± 10.4 (*n* = *4*)	11.1 ± 4.7 WU m^2^ (*n* = *4*)	11 (*n = 1*)	3.2 ± 0.05 (*n* = *3*)	100% (4/4) paediatric cases responsive to acute vasodilator challenge (but adult relatives with the same variant were not)	–
*EIF2AK4* (GCN2)	609280	PVOD/PCH (91%); IPAH (9%)	AR	Some reports of biallelic *EIF2AK4* variants in clinically diagnosed paediatric PAH	11	15.1 ± 2.0 (*n* = *8*)	52.3 ± 20.1 (*n* = *7*)	11.3 ± 6.1 WU m^2^ (*n* = *3*)	6.3 ± 1.7 (*n* = *3*)	2.6 ± 1.3 (*n* = *3*)	Use of vasodilators associated with life-threatening pulmonary oedema	58, 62
*ENG*	131195	PAH-HHT (60%); IPAH (20%); APAH-CHD (20%)	AD	HHT type 1 (telangiectasia and PAVMs, epistaxis)	5	8.2 ± 6.7 (*n* = *5*)	46.3 ± 5.0 (*n* = *3*)	10.5 ± 4.3 WU (*n* = *3*)	7.3 ± 3.3 (*n* = *3*)	3.2 ± 0.5 (*n* = *3*)	–	[10]
*GDF2* (BMP9)	605120	IPAH (78%); PAH-HHT (22%)	AD; AR	>HHT type 5 (telangiectasia, AVMs, epistaxis); PAVMs >Homozygous variants are also associated with ‘HHT-like’ facial telangiectases and diffuse PAVMs	9	8.4 ± 5.6 (*n* = *7*)	58.8 ± 21.3 (*n* = *6*)	13.4 ± 10.9 WU (*n = 6*)	7.0 ± 0.8 (*n* = *3*)	4.5 ± 2.0 (*n* = *6*)	–	15••, 17•, 19, 20
*KCNK3* (TASK1)	603220	IPAH (57%); HPAH (43%)	AD	–	9	6.4 ± 3.8 (*n* = *6*)	97.0 ± 10.0 (*n* = *2*)	44 ± 8.0 WU (*n* = *2*)	10 (*n = 1*)	2.4 ± 0.3 (*n* = *2*)	–	26, 27, 29
*NOTCH3*	600276	IPAH	AD	>Heterozygous variants are a major cause of CADASIL >Gene is also linked to lateral meningocele syndrome	4	4.0 ± 0 (*n* = *2*)	68.5 ± 1.5 (*n* = *2*)	nd	12 (*n* = *1*)	2.9 (*n* = *1*)	–	–
*SMAD9* (SMAD8)	603295	IPAH (43%); APAH-CHD (43%); HPAH (14%)	AD	–	7	5.7 ± 4.6 (*n* = *7*)	70.0 ± 17 (*n* = *2*)	24.9 ± 12.5 WU m^2^ (*n* = *2*)	6.0 ± 0 (*n* = *2*)	3.2 ± 1.1 (*n* = *2*)	–	–
*SOX17*	610928	APAH-CHD (63%); IPAH (25%); HPAH (12%)	AD	>Strongly linked to APAH-CHD >Gene may also be associated with vesicoureteral reflux	15	4.9 ± 3.6 (*n* = *18*)	34.5 ± 2.5 (*n* = *2*)	8.0 ± 1.0 WU (*n* = *2*)	5.5 ± 0.5 (*n* = *2*)	nd	–	40•, 44
*TBX4*	601719	IPAH (33%); APAH-CHD (31%); PAH-SPS (29%); HPAH (8%); other (4%)	AD	>Small patella syndrome with/without PAH >Abnormal distal lung development >Cardiac and skeletal malformations >Copy number variants (CNVs) are more commonly associated with developmental delay	60 (*incl. 10 cases with PPHN*)	4.3 ± 4.8 (*n* = *52*)	59.8 ± 24.4 (*n* = *24*)	17.5 ± 15.5 WU m^2^ (*n* = *25*)	9.5 ± 4.5 (*n* = *18*)	3.9 ± 2.2 (*n* = *24*)	35% (8/23) patients demonstrate partial vasoreactivity	25••, 51•, 52, 53

Table summarising the clinical features, onset and severity of disease related to genes reported to cause paediatric PAH. Haemodynamic data are provided as mean ± standard deviation at diagnosis for *n* patients (includes some related individuals). AVMs: arteriovenous malformations; CADASIL: cerebral autosomal dominant arteriopathy with subcortical infarcts and leukoencephalopathy; CI: cardiac index; mPAP: mean pulmonary artery pressure; mPWP: mean pulmonary wedge pressure; nd: no data available; OMIM: Online Mendelian Inheritance in Man (https://www.omim.org); PAVMs: pulmonary arteriovenous malformations; PVR(i): pulmonary vascular resistance (index).

Children with pathogenic variants in *BMPR2* or *ACVRL1*, with or without HHT, have poor clinical outcomes by comparison to noncarriers, typically experiencing a marked limitation of physical activity (WHO functional class III) within 3 years of diagnosis, highlighting the need for rapid mutation detection and treatment 6, 10. Of note, 14–16% of deleterious variation in *BMPR2* comprises structural variants that require additional analysis beyond targeted exonic sequencing 7, 8. Whilst this has traditionally been conducted by multiplex ligation-dependent probe amplification, more innovative technologies include the use of mate-pair sequencing and MinION sequencing to comprehensively screen for intronic variants 11, 12.

Additional risk factors impacting BMP/TGF-β signalling in PAH include SMAD8 (*SMAD9*) and caveolin-1 (*CAV1*), identified by candidate gene screening and whole-exome sequencing (WES), respectively. SMAD8 is a BMP-specific signalling intermediary whereas CAV1 plays an important role in regulation of the TGF-β signalling system. Heterozygous variants in these genes have been reported to cause PAH in some paediatric-onset cases [9]. *CAV1* may additionally be associated with a neonatal onset lipodystrophy syndrome [13].

### BMP ligands in pulmonary arterial hypertension pathogenesis

BMP9 and BMP10 are circulating ligands that specifically activate the ALK1/BMPR2 receptor complex. Rare heterozygous variants in the BMP9 (*GDF2*) gene are significantly enriched in adult-onset disease, based on exome-wide gene burden testing, and represent between 0.8% and 6.6% of all PAH cases with over 60 pathogenic variants reported to date 14, 15••, 16, 17•, 18. Of interest, the pathophysiology in children indicates a potential semi-dominant inheritance for this gene in PAH; two reports have identified biallelic variants leading to early-onset disease, including one case with PAH-HHT 19, 20. Evidence for a role of heterozygous *BMP10* variation is also starting to emerge with four likely deleterious variants described, of which two were detected in childhood-onset PAH 17•, 18, 21. Of interest, plasma levels of both ligands are markedly reduced in *GDF2* mutation carriers, likely due to impaired cellular processing and secretion 15••, 18, 19.

## Pathogenic variation in non-BMP/TGF-β pathway genes

### Identification of ATP13A3 variants in childhood-onset cases

*ATP13A3* encodes a member of the P5B subfamily of integral membrane ATPases, most recently reported to be a polyamine transporter [22]. Polyamines are polycations with numerous functions in cellular processes that include a cardioprotective role. The *ATP13A3* gene was first identified as a risk factor for PAH by large-scale whole-genome sequencing. Likely pathogenic heterozygous variants were significantly enriched in adult PAH cases and were predicted to disrupt ATPase activity [14]. Subsequent WES analysis in a paediatric cohort detected a novel *ATP13A3* missense variant in a child with PAH and atrial septal defect (ASD) [21]. Most recently, Machado et al. performed next-generation sequencing (NGS) in three families with childhood-onset PAH [23]. Analysis of *ATP13A3* identified one homozygous and two compound heterozygous variants, of which p.Met850Ilefs*13 had been previously reported as heterozygous in an adult-onset case [16]. Whilst the majority of variants are anticipated to be null alleles, a nonsense variant in the final exon was predicted to evade nonsense-mediated decay, likely leading to the formation of an abnormal protein product. This study supports a model of dose-dependent, semi-dominant inheritance for this gene, wherein heterozygous variants predominantly underlie adult-onset disease and deleterious biallelic *ATP13A3* variants cause a severe, autosomal recessive (AR) form of paediatric PAH [23].

### Potassium ion transporters implicated in pulmonary arterial hypertension

*KCNK3* was one of the first non-BMP pathway genes to be identified as a novel cause of autosomal dominant (AD) PAH by WES in a predominantly adult-onset I/HPAH cohort. The gene encodes a hypoxia-sensitive potassium channel involved in the regulation of the resting membrane potential of pulmonary arterial smooth muscle cells and pulmonary vascular tone [24]. While its contribution to paediatric disease remains limited 9•, 25••, Navas Tejedor et al. have reported a homozygous missense variant in a child diagnosed with an aggressive form of HPAH at two months of age [26]. The variant is located in a highly conserved amino acid and has been demonstrated to significantly reduce channel current [27]. A loss-of-function mechanism of disease is supported by the *Kcnk3* rat model, which demonstrates pulmonary vascular abnormalities consistent with PAH [28].

*ABCC8* is a member of the ABC family which encodes sulfonylurea receptor 1 (SUR1) protein, a regulatory subunit of the K_ATP_ channel that controls channel function and potassium ion (K^+^) transport [29]. Using WES in a cohort of childhood- and adult-onset PAH patients, Bohnen et al. identified a novel, predicted deleterious missense variant in a child with IPAH [30]. Screening of a larger cohort detected 11 further likely pathogenic variants, of which six were missense variants in paediatric cases with IPAH, HPAH or APAH-CHD. All the missense variants lie in critical functional domains and patch-clamp experiments confirmed a loss of ATP-sensitive potassium channel function, which was rescued by the ABCC8 activator diazoxide [30]. This gene has recently been independently replicated with the detection of likely damaging heterozygous variants in>30 PAH patients, including six children diagnosed between 1 and 18 years 16, 21, 25••.

The identification of loss-of-function variants of *KCNK3* and *ABCC8* in PAH provides novel exploratory avenues for molecular pathogenesis and may point to complementary or redundant functions of KCNK3 and K_ATP_ [30]. Variants in *KCNA5* have also been implicated as potential modifiers in PAH pathogenesis, including in an early-onset case of severe PAH with a pathogenic *BMPR2* variant [31], but this has not yet been convincingly replicated. The implication of potassium channels in the pathophysiology of PAH is discussed elsewhere [29].

### Recent gene discoveries

Recent novel gene discoveries include independent reports of heterozygous loss-of-function variants in kinase insert domain receptor, *KDR* which is characterised by PAH with low diffusing capacity for carbon monoxide (DLCO) 32, 33, 34. Additionally, two substantive PAH study cohorts comprising patients from the National Biological Sample and Data Repository for PAH (PAH Biobank), Columbia University Irving Medical Center, and the UK NIHR BioResource Rare Diseases Study have used NGS-based gene burden testing for novel gene detection 16, 34. Each study identified likely deleterious variants in two genes, namely tissue kallikrein 1 (*KLK1*) and gamma glutamyl carboxylase (*GGCX*) [16], fibulin 2 (*FBLN2*) and platelet-derived growth factor D (*PDGFD*) [34]. Of note, whilst only three paediatric variant carriers were detected in these genes, child-parent trio analyses highlighted a significant enrichment of predicted damaging *de novo* variants in additional genes, potentially explaining ~15% of paediatric PAH cases in their cohort. Given the limited time since initial discovery, further studies will be required to fully elucidate the role of these newly described genes in the pathogenesis of paediatric PAH.

## Associated and syndromic forms of pulmonary arterial hypertension

A particular consideration in neonatal- and paediatric-onset pulmonary hypertension (PH) is the presence of complex comorbidities, often influenced by early developmental impacts on lung growth. The most common cause of transient PAH in neonates is persistent pulmonary hypertension of the newborn (PPHN), a failure of immediate postnatal cardiopulmonary transition with an annual incidence of 30.1 cases/million 1, 35. The genetic aetiology of PPHN is unclear; although potential susceptibility variants have been recently reported in *CPS1*, *NOTCH3*, *SMAD9* and the hypoxia-related genes *TTLL3* and *ITGAM*, these remain to be independently validated 36, 37.

PH is also seen in children with connective tissue disease (APAH-CTD) or developmental lung diseases such as alveolar capillary dysplasia, bronchopulmonary dysplasia and congenital diaphragmatic hernia 1, 38. Less frequent syndromic causes of PAH include ischiocoxopodopatellar syndrome (see TBX4 below) and Adams–Oliver syndrome [39].

### Pulmonary arterial hypertension and congenital heart disease

Childhood forms of PAH are frequently associated with congenital heart disease (CHD), which forms Group 1.4.4 of the sixth World Symposium on Pulmonary Hypertension classification (Table 1). Germline variants in established risk genes such as *BMPR2*, *ENG*, *SMAD9*, *CAV1* and *BMP10* have been linked to progressive PAH in children or young adults 9•, 16, 17•. In 2018, *SOX17* was identified as a PAH risk gene by gene burden testing in an IPAH cohort [14]. Initial analysis of a family harbouring a p.Tyr137* nonsense mutation suggested that this variant may underlie an early-onset form of PAH associated with an ASD. Subsequently, WES in an independent cohort of 256 cases confirmed *SOX17* as a major cause of APAH-CHD, with the identification of rare deleterious missense variants in 9/13 paediatric cases [40]. Other reports of childhood-onset disease include four likely pathogenic variants in a cohort of 2572 PAH cases from a North American PAH biobank [16], plus a Japanese case diagnosed at two years of age with IPAH and patent foramen ovale, who carried heterozygous variants in *SOX17* and *TBX4*, both inherited from an unaffected parent [41].

*SOX17* encodes an endothelial transcription factor belonging to the SRY-related HMG box gene family involved in vascular development. SOX17 and its homologue SOX18 are crucial during embryonic development in processes such as angiogenesis, arterial specification and pulmonary vascular morphogenesis [42]. Pathogenic variants identified in PAH predict loss-of-function due to haploinsufficiency, in turn impacting SOX17-related pathways such as Wnt/β-catenin and Notch signalling 43, 44. Of interest, variants in both *NOTCH1* and *NOTCH3* have been described in childhood-onset PAH. NOTCH1 haploinsufficiency is a major cause of both nonsyndromic CHD and Adams–Oliver syndrome; the identification of rare *NOTCH1* variants in children with APAH-CHD would therefore be consistent with a secondary form of PAH, albeit in relatively few cases 34, 45, 46. By contrast, although dysregulated NOTCH3 signalling is well documented to play an important role during vascular remodelling in PAH, the evidence for a deleterious impact of inherited *NOTCH3* variation remains limited [47].

### TBX4 in childhood pulmonary arterial hypertension

The *TBX4* gene encodes a member of the T-box family of transcription factors that, together with *TBX5*, has an essential role in development of the limbs and respiratory system 48, 49. Both genes are co-expressed throughout the pulmonary mesenchyme and TBX4 has recently been shown to positively regulate phospho-SMAD1/5, indicating potential crosstalk with BMP signalling [50]. Heterozygous *TBX4* variants cause ischiocoxopodopatellar syndrome (ICPPS; OMIM #147891), also known as small patella syndrome, whilst homozygous loss-of-function variants underlie posterior amelia with pelvic and pulmonary hypoplasia syndrome (PAPPAS; OMIM #601360). Heterozygous *TBX4*-containing deletions or likely pathogenic *TBX4* variants have been confirmed as a substantial cause of paediatric PAH, accounting for up to 8% of familial and idiopathic disease, with or without ICPPS 6, 25••. Of note, the mean age-of-onset in heterozygous *TBX4* carriers is younger than *BMPR2* carriers, demonstrating a significant enrichment in childhood- versus adult-onset cases 16, 25•• (Table 2). Genetic variation of *TBX4* ranges from single nucleotide variants to large (>2 Mb) deletions, encompassing multiple genes. The latter are more commonly associated with developmental delay and additional neurological and psychomotor defects, whereas PAH is typically caused by protein-truncating or deleterious missense variants 51•, 52. Whilst early reports indicated a milder presentation in some PAH patients, it is now recognised that the clinical phenotypes associated with *TBX4* disruption represent a broad spectrum, ranging from transient neonatal PH to severe developmental lung disorders and progressive or biphasic PH, which may be associated with skeletal, cardiac and/or neurological anomalies. Moreover, *TBX4* variants underlie a wide spectrum of clinicopathological outcomes. Neonatal respiratory failure has been reported due to lethal lung hypoplasia, such as acinar dysplasia or congenital alveolar dysplasia. By contrast, chronic PH can be diagnosed later in infancy and may recur in children previously recovered from PPHN 51•, 52, 53, 54. Taken together, the concept of a ‘*TBX4* syndrome’ characterised by severe pre-capillary PH points to a complex aetiology and disease progression that should be supported by regular cardiopulmonary assessment and multisystem imaging 9•, 55.

### Pulmonary veno-occlusive disease and pulmonary capillary haemangiomatosis

Pulmonary veno-occlusive disease (PVOD2; OMIM #234810) and pulmonary capillary haemangiomatosis (PCH) are clinically similar subtypes of PAH with AR inheritance. Although histologically distinct, PCH and PVOD include venous and/or capillary abnormalities that are now clinically classified under PAH Group 1.6 [2] (Table 1). Despite most cases of PVOD/PCH being acquired, biallelic variants in the eukaryotic translation initiation factor 2 alpha kinase 4 (*EIF2AK4*) gene have been described in children and young adults 6, 7, 56. The majority of variants are predicted to lead to premature protein truncation, consistent with a loss-of-function mechanism of disease. Experimental models of EIF2AK4 loss-of-function have been reported to negatively regulate BMP-dependent SMAD1/5/8 signalling and proliferation of pulmonary arterial endothelial cells was reversed by exogenous BMP9, highlighting a potential therapeutic avenue [57].

*EIF2AK4* variants are associated with low DLCO, which is a characteristic feature of PVOD/PCH [58]. Importantly, PVOD and PCH are often misclassified as HPAH or IPAH as accurate diagnosis requires microscopic examination of lung tissue biopsy, which may be unsafe in some patients. Of note, some H/IPAH patients have been described with heterozygous variants in *EIF2AK4*. However, it remains unclear whether these variants are a rare cause of AD PAH or potential genetic modifiers. Cases of clinically diagnosed PAH in whom biallelic *EIF2AK4* variants have been detected are likely to represent previously misdiagnosed PVOD/PCH 17•, 56. Although some patients may respond well to PAH-targeted therapy 59, 60, pulmonary vasodilator agents can cause fatal pulmonary oedema in PVOD/PCH so should be used with caution. Genetic testing is therefore highly recommended to support accurate diagnosis; given the rapid disease progression in paediatric patients, an early referral for lung transplantation would also be beneficial 61, 62.

## Conclusions

**Figure 2:**
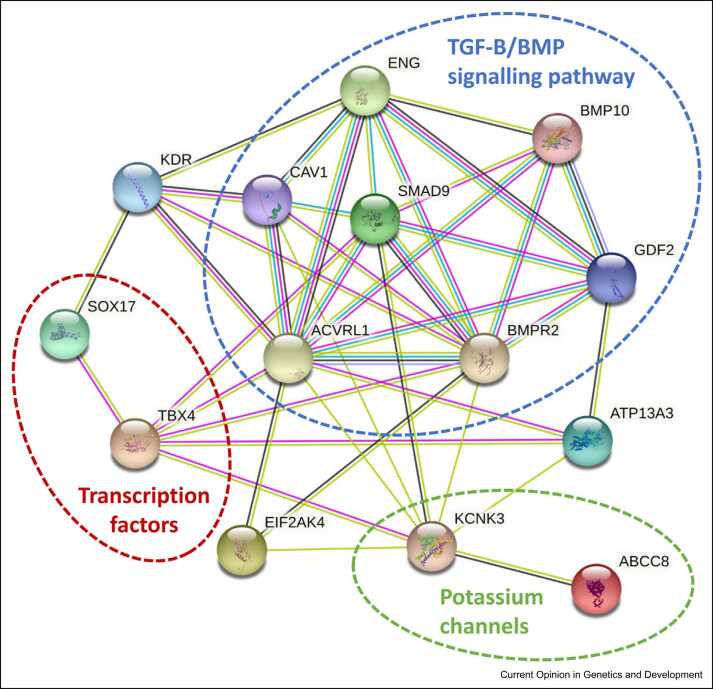
Genes underlying PAH in children. Schematic highlighting independently validated genes and protein clusters in paediatric PAH. Connecting lines denote indicative relationships between proteins, for example, based on text-mining, database, co-expression, and protein interaction data. Multiple lines indicate stronger evidence for a causal relationship. Network image was created using STRING (https://string-db.org/cgi/input).

Recent advances from large-scale sequencing studies have identified multiple new risk genes in PAH (Figure 2). Whereas pathogenic *BMPR2* variation remains the predominant cause, the vast majority of newly reported genes each describe less than 3% of paediatric cases (Table 2). A notable exception is *TBX4*, which contributes to 7.7% of IPAH and 4.9% of APAH-CHD cases in children, highlighting a significant divergence between adult- and childhood-onset disease [9]. Of interest, the examination of severe, early-onset cases has also highlighted a new paradigm of semi-dominant inheritance for some genes, namely *ATP13A3* and *GDF2*, which may impact genetic counselling in at-risk individuals.

While these novel findings provide unique opportunities, challenges remain. First, independent validation of new gene discoveries is critical to ensure robust interpretation of identified variants in a clinical context. This has important implications for the design of molecular diagnostic testing panels to best drive clinical management decisions. Second, the phenotypic heterogeneity in paediatric PH may complicate interpretations of pathogenicity. Combining clinical and molecular genetic data into a detailed investigation of genotype-phenotype correlations will provide greater confidence for diagnosis and disease prognosis.

Finally, this expanded genetic architecture highlights new molecular pathways important in the pathogenesis of paediatric PAH. Elucidating the molecular networks that link established and recently identified risk pathways will ultimately provide mechanistic insights to support future pre-clinical testing of repurposed or novel therapeutics.

## Funding

This work was supported by the Springboard Scheme Funders, namely the 10.13039/501100000691Academy of Medical Sciences (AMS), the 10.13039/100004440Wellcome Trust, the Government Department of Business, Energy and Industrial Strategy (BEIS), the 10.13039/501100000274British Heart Foundation and 10.13039/501100000361Diabetes UK [SBF005\1115]. For the purpose of Open Access, the author has applied a CC BY public copyright licence to any Author Accepted Manuscript (AAM) version arising from this submission.

## Author contributions

Fatima Taha: Investigation, Visualization, Writing – original draft; Laura Southgate: Conceptualization, Formal analysis, Visualization, Writing – original draft, Writing – review & editing.

## Conflict of interest statement

The authors declare no conflict of interest.

## References and recommended reading

Papers of particular interest, published within the period of review, have been highlighted as:

**•** of special interest

**••** of outstanding interest.
